# Prediction of recurrent heart failure hospitalizations and mortality using the echocardiographic Killip score

**DOI:** 10.1007/s00392-024-02473-8

**Published:** 2024-06-03

**Authors:** Yoav Granot, Yuval Meir, Michal Laufer Perl, Assi Milwidsky, Ben Sadeh, Orly Ran Sapir, Adva Trabelsi, Shmuel Banai, Yan Toplisky, Ofer Havakuk

**Affiliations:** 1 Department of Cardiology, Tel Aviv Medical Center, 6 Weizmann Street, 6423906 Tel Aviv, Israel; 2 Faculty of Medicine, Tel Aviv University, Tel Aviv, Israel; 3 Zena and Michael A. Wiener Cardiovascular Institute, Icahn School of Medicine at Mount Sinai, New York, NY USA; 4 Division of Preventive Cardiology, Department of Cardiovascular Medicine, Mayo Clinic, Rochester, MN USA

**Keywords:** Heart failure readmissions, Killip score, Echocardiography, Prediction

## Abstract

**Aim:**

Examine the performance of a simple echocardiographic "Killip score" (eKillip) in predicting heart failure (HF) hospitalizations and mortality after index event of decompensated HF hospitalization.

**Methods:**

HF patients hospitalized at our facility between 03/2019–03/2021 who underwent an echocardiography during their index admission were included in this retrospective analysis. The cohort was divided into 4 classes of eKillip according to: stroke volume index (SVI) < 35ml/m^2^ > and E/E' ratio < 15 > . An eKillip Class I was defined as SVI ≥ 35ml/m^2^ and E/E' ≤ 15 and was used as reference.

**Results:**

Included 751 patients, median age 78.1 (IQR 69.3–86) years, 59% men, left ventricular ejection fraction 45 (IQR 30–60)%, brain natriuretic peptide levels 634 (IQR 331–1222)pg/ml. Compared with eKillip Class I, a graded increase in the combined endpoint of 30-day mortality and rehospitalizations rates was noted: (Class II: HR 1.77, CI 0.95–3.33, p = 0.07; Class III: HR 1.94, CI 1.05–3.6, p = 0.034; Class IV: HR 2.9, CI 1.64–5.13, p < 0.001 respectively), which overall persisted after correction for clinical (Class II: HR 1.682, CI 0.9–3.15, p = 0.105; Class III: HR 2.104, CI 1.13–3.9, p = 0.019; Class IV: HR 2.74, CI 1.54–4.85, p = 0.001 respectively) or echocardiographic parameters (Class II: HR 1.92, CI 1.02–3.63, p = 0.045; Class III: HR 1.54, CI 0.81–2.95, p = 0.189; Class IV: HR 2.04, CI 1.1–3.76, p = 0.023 respectively). Specifically, the eKillip Class IV group comprised one-third of the patient population and persistently showed increased risk of 30-day HF hospitalizations or mortality following multivariate analysis.

**Conclusion:**

A simple echocardiographic score can assist identifying high-risk decompensated HF patients for recurrent hospitalizations and mortality.

**Supplementary Information:**

The online version contains supplementary material available at 10.1007/s00392-024-02473-8.

## Introduction

The burden of heart failure (HF) continues to rise and besides its significant mortality risk, the medical costs spent on HF hospitalizations are expected to increase from $21 billion in 2012 to $53 billion in 2030 [1]. Nevertheless, not all HF patients poses a similar risk for rehospitalizations or mortality and there is an urgent need for simple and reliable tools which will assist with differentiating high vs low risk patients.

In 1967, Killip and Kimball [2] described a clinical tool for predicting mortality risk in hospitalized acute myocardial infarction patients. Four risk classes were described according to the presence of pulmonary congestion ("dry/wet") and peripheral perfusion ("warm/cold"), where class IV ("wet and cold") was associated with worst prognosis. This score was later repeatedly validated [3–6].

We have developed an echocardiographic Killip score (eKillip) according to echocardiographic filling pressures (as a surrogate for pulmonary congestion) and stroke volume index (SVI) (as a surrogate for peripheral perfusion) and demonstrated its ability in risk stratifying a large group of both admitted and ambulatory cardiovascular patients [7]. Previous studies had shown that echocardiographic derived hemodynamic profiles can predict outcome in ambulatory HF patients [8, 9].

Here we attempted to examine the ability of a refined eKillip score in predicting 30-day recurrent HF hospitalizations and mortality among hospitalized decompensated HF patients.

## Methods

### Population

This is a retrospective analysis of all adult HF patients admitted to our facility between 03/2019 and 03/2021 due to decompensated HF who underwent an echocardiographic exam during their index admission. Files were reviewed and HF diagnosis was based upon accepted criteria including: signs and symptoms and structural cardiac changes (i.e. left ventricular ejection fraction (LVEF) < 40% or increased LV wall thickness / left atria (LA) size or impaired diastolic function) together with brain natriuretic peptide (BNP) levels > 100pg/ml [10]. The initial cohort included 860 patients. Patients in whom diastolic function could not be reliably assessed (e.g. prosthetic mitral valve, mitral annular calcification) (n = 58) and those in whom echocardiography data were missing (n = 51) were excluded from the study. No other exclusion criteria were applied. The study was approved by the Institutional Review Board.

### Clinical data and outcomes

Baseline characteristics including age, sex, comorbidities and medications were extracted from the electronic health record. Thirty-day mortality or HF hospitalizations were retrieved from the Institutional electronic health records. The date of mortality was automatically updated from the Ministry of Health and retrieved by a unique identification number while keeping patients' anonymity.

### Echocardiography

A comprehensive echocardiography was performed in accordance with contemporary guidelines [11]. Specifically, pulsed-wave Doppler was performed in the apical 4-chamber view to obtain mitral inflow velocities to assess LV filling. A 1-mm to 3-mm sample volume was placed between the mitral leaflet tips at end-expiration and during diastole after optimizing spectral gain, wall filter settings, and setting sweep speeds of 100 mm/s. Measurements of mitral inflow included the peak early filling (E wave) and late diastolic filling (A wave) velocities, the E/A ratio, and deceleration time of early filling velocity. Early diastolic mitral annular velocities (E') were measured in the apical 4-chamber view. The E’ was measured from septal and lateral annulus. The ratio of peak E to peak E' was calculated (E/E' ratio) from the average of ≥ 3 and ≥ 6 cardiac cycles in sinus rhythm and atrial fibrillation (AF) respectively. Stroke volume was calculated as the product of LV outflow tract area and the time-velocity integral of the aortic flow velocity and was indexed to body surface area (BSA). Pulmonary artery systolic pressure (PASP) was evaluated according to the combination of inferior vena cava size and collapsibility and tricuspid regurgitation pressure gradient (TRPG). RV size and function assessment was based on multiple views of the RV. An integrative qualitative grading was formulated by the physician responsible for the echocardiographic study, that incorporates visual assessment and quantitatively by assessment of tricuspid annular plane systolic excursion (TAPSE) and DTI-derived tricuspid lateral annular systolic velocity wave (S'). Patients with moderate or severe RV dilatation or dysfunction were grouped together for the statistical analysis.

#### The eKillip score

To produce a simple and straightforward tool, the cohort was divided into 4 classes according to a combination of 2 echocardiograhic parameters—SVI and E/E' ratio:

- Class I – E/E' ≤ 15 and SVI ≥ 35ml/m^2^
- Class II – E/E' > 15 and SVI ≥ 35ml/m^2^
- Class III – E/E' ≤ 15 and SVI < 35ml/m^2^
- Class IV – E/E' > 15 and SVI < 35ml/m^2^

We used an E/E’ cutoff value (> 15) that is relatively specific for elevated left sided filling pressure when compared with invasive measurements [12], and has been shown in multiple studies to correlate with clinical outcomes [13, 14].

Of note, despite the potential effect of fluid accumulation on BSA and consequently on SVI measurement, we chose to use SVI and not uncorrected SV in accordance with contemporary guidelines [11], but also due to the minor potential effect of it on SVI measurement [15].

Both E/e’ and SVI (15 and 35ml/m^2^ respectively) cutoff values were chosen based on previous published data and with accordance with current guideline.

### Statistical methods

Categorical variables were reported as numbers and percentages, and continuous variables were reported as means and standard deviations or medians and interquartile ranges (IQRs), as appropriate. Continuous variables were tested for normal distribution using histograms, Q-Q Plots and normality tests (Shapiro–Wilk). Continuous variables were compared between groups using independent samples t- test or Mann–Whitney test and categorical variables were compared using Chi-square test or Fisher's exact test.

Thirty-day mortality or HF hospitalizations was assessed using a Cox regression model. In order to minimize the impact of patients that may not have been fully medically optimized during their index admission, we did not include very early re-admissions (up to 10 days from discharge) into the analysis.

We also performed an adjusted regression analysis, with the following variables included:

#### Clinical variables

Age, Sex, presence of chronic kidney disease (i.e. glomerular filtration rate < 60ml/min), hypertension, diabetes mellitus, coronary artery disease and AF, hemoglobin levels and guideline-directed HF medication use at admission and at discharge.

#### Echocardiographic variables

LVEF, LV end systolic and end diastolic diameter, E/A ratio, LA size, TRPG, right ventricular size and function.

As the number on patient in each group was relatively low, we could not adjust the analysis for both clinical and echocardiographic variable while maintaining a ratio of a minimum of 10 events per variable.

A two-tailed p-value less than 0.05 was considered as statistically significant. All statistical analyses were performed with SPSS (IBM Corp. IBM SPSS Statistics for Windows, Version 24.0. Armonk, NY: IBM Corp).

## Results

The final cohort included 751 patients, 445 men (59%), median age 78.1 (IQR 69.3–86) years. Echocardiography study was performed within 1.7 days from admission (IQR 0.9–3.0 days) and the patient admission length of stay was 6.1 days (IQR 3.7–11.1 days).

Coronary artery disease was reported in 61% and 41% had AF. BNP levels were 634 (IQR 331–1222)pg/ml and creatinine levels were 1.3 (IQR 0.9–1.8)mg/dl (Table 1). Statistically significant between-group differences were noted in the prevalence of HTN, DM and AF (Table 1). Median LVEF was 45 (IQR 30–60)%, (LVEF < 40% in 43.5% of patients). Median SVI 32.5 (IQR 26–40.2)ml/m^2^ and E/E' 16 (IQR 12.5–21) (Table 2). The overall 30-day readmission rates were 14.9% and the overall 30-day mortality rates were 7.1%.

**Table 1:** Epidemiologic characteristics, underlying disease and laboratory values according to eKillip class

	ALL (*n* = 751)	Class I (*n* = 125)	Class II (*n* = 186)	Class III (*n* = 180)	Class IV (*n* = 260)	*P*
Age	78.1 (69.3–86)	77.8 (70.3–85.3)	81.1 (71.8–88.1)	76.1 (67.9–84.1)	78.7 (68.9–86.3)	0.015
Male sex- no. (%)	445 (59)	76 (61)	84 (45)	103 (57)	182 (70)	< 0.001
BSA, m^2^ (IQR)	1.9 (1.7–2)	1.9 (1.7–2)	1.8 (1.7–1.9)	1.9 (1.7–2)	1.9 (1.7–2)	0.025
Systolic BP, mmHg (IQR)	134 (117–152)	135 (120.5–153.5)	142 (125.8–155)	130.5 (114–150)	129 (114–146)	< 0.001
Diastolic BP, mmHg (IQR)	70 (60–83)	70 (59.5–83)	66 (56–78)	73 (62–86)	71 (61–83)	< 0.001
Heart rate, bpm (IQR)	71 (62–83)	67 (59.5–77.5)	65 (59.8–74)	78 (67–90)	74 (66–88)	< 0.001
Medical history- no. (%):						
Hypertension	590 (79)	96 (77)	164 (88)	133 (74)	197 (76)	0.003
DM	353 (47)	54 (43)	99 (53)	71 (39)	129 (50)	0.037
AF	308 (41)	40 (32)	74 (40)	87 (48)	107 (41)	0.04
IHD	455 (61)	73 (58)	108 (58)	103 (57)	171 (66)	0.21
Pacemaker/ICD	74 (10)	9 (7)	17 (9)	17 (9)	31 (12)	0.496
CVA/TIA	125 (17)	21 (17)	37 (20)	23 (13)	44 (17)	0.338
CKD	232 (31)	37 (30)	68 (37)	37 (21)	90 (35)	0.004
Hyperlipidemia	408 (54)	59 (47)	113 (61)	91 (51)	145 (56)	0.075
PVD	86 (11)	14 (11)	24 (13)	17 (9)	31 (12)	0.761
COPD	106 (14)	22 (18)	29 (16)	24 (13)	31 (12)	0.439
Malignancy	193 (26)	33 (26)	55 (30)	43 (24)	62 (24)	0.519
Medications at discharge: no.(%)						
Diuretics	630 (84)	102 (82)	160 (86)	151 (84)	217 (84)	0.774
Beta blocker	568 (76)	88 (71)	136 (73)	148 (82)	196 (76)	0.08
ACEi/ARB/ARNI	447 (60)	71 (57)	103 (55)	117 (65)	156 (60)	0.261
MRA	232 (31)	30 (24)	39 (21)	59 (33)	104 (40)	< 0.001
SGLT2i	77 (10)	7 (6)	18 (10)	16 (9)	36 (14)	0.069
Laboratory						
Serum creatinine, mg/dl (IQR)	1.3 (0.9–1.8)	1.1 (0.9–1.8)	1.3 (0.9–1.8)	1.1 (0.9–1.6)	1.3 (1–1.9)	0.015
Hemoglobin, g/dl (IQR)	11.6 (10.2–13.1)	11.7 (10.6–13.3)	10.7 (9.4–12.2)	12.3 (10.9–13.6)	11.6 (10.5–13.3)	< 0.001
BNP, pg/ml (IQR)	634 (331.5–1222.3)	480 (260.8–1031.8)	516 (279–1093)	693 (410–1170)	798.5 (418–1323)	0.002

Continuous variables are expressed as median and interquartile range (IQR)

*ACEi* angiotensin-converting enzyme inhibitor, *AF* atrial fibrillation, *ARB* angiotensin receptor blocker, *ARNI* angiotensin receptor neprilysin inhibitor, *BNP* brain natriuretic peptide, *BP* blood pressure; bpm, beat per minute, *BSA* body surface area, *CKD* chronic kidney disease, *COPD* chronic obstructive pulmonary disease, *CVA* cerebral vascular accident, *DM* diabetes mellitus, *ICD* implantable cardioverter defibrillator, *IHD* ischemic heart disease, *MRA* mineralocorticoid receptor antagonist, *PVD* peripheral vascular disease, *SGLT2i* sodium-glucose contransporter-2 inhibitor, *TIA* transient ischemic attack

*P* values refer to the difference between one and any of the remaining three groups

**Table 2:** Echocardiographic characteristics according to eKillip class

		ALL (*n* = 751)	Class I (*n* = 125)	Class II (*n* = 186)	Class III (*n* = 180)	Class IV (*n* = 260)	*P*
EF, %		45 (30–60)	55 (40–60)	55 (45–60)	40 (30–55)	35 (30–50)	< 0.001
LV EDD, mm		51 (46–58)	51 (46.5–58)	50 (46–55)	52 (45–59)	53 (45.5–60)	0.067
LV ESD, mm		36 (30–46)	34 (29–42)	33 (28–39.8)	38 (30–48)	41 (30–50)	< 0.001
LAVI, mL/m^2^		50 (41–60)	46.8 (38.4–56.5)	51 (41–62.5)	48.9 (39–59.6)	52 (43–60.8)	0.025
LV SVI, mL/m^2^		32.5 (26–40.2)	41.1 (38.3–46.6)	42.2 (38.7–48.9)	26.8 (23.5–31.3)	27 (23.2–31.1)	< 0.001
E/A ratio		1.3 (0.9–2)	1 (0.7–1.5)	1.3 (1–1.7)	1.1 (0.7–1.8)	1.6 (1.2–2.6)	< 0.001
DT, msec		172 (137–211)	193 (148–246)	180 (148–218)	167 (134.3–201)	152 (123.8–193)	< 0.001
Average E/E'		16 (12.5–21)	11.8 (10–13)	20 (17–24.1)	12 (9.3–13)	19.9 (17–24)	< 0.001
TAPSE, mm		2.5 (1.7–16)	2.8 (2–20)	2.7 (2–19)	3.2 (1.8–16)	2 (1.5–13.3)	< 0.001
TRPG, mmHg		37 (29–46)	39 (27–49.5)	40 (33–47)	32 (24–42)	39 (31–47.8)	< 0.001
RA pressure, mmHg	5	242 (36)	59 (55)	69 (42)	53 (34)	61 (25)	< 0.001
	10	154 (23)	25 (23)	43 (26)	32 (21)	54 (22)	
	15	126 (19)	11 (10)	32 (20)	26 (17)	57 (24)	
	20	145 (21)	12 (11)	20 (12)	43 (28)	70 (29)	
RV size	Normal	482 (64)	95 (76)	144 (77)	103 (57)	140 (54)	< 0.001
	Mild	188 (25)	23 (18)	34 (18)	46 (26)	85 (33)	
	Moderate – Severe	81 (11)	7 (6)	8 (4)	31 (17)	35 (14)	
RV function	Normal	521 (69)	109 (87)	157 (84)	105 (58)	150 (58)	< 0.001
	Mild	147 (20)	12 (10)	23 (12)	53 (29)	59 (23)	
	Moderate—Severe	83 (11)	4 (3)	6 (3)	22 (12)	50 (20)	

Data are expressed as median and interquartile range (IQR)

*DT* deceleration time, *EDD* end-diastolic diameter, *EF* ejection fraction, *ESD* end-systolic diameter, *LV* left ventricular, *RA* right atrial, *RV* right ventricle, *TAPSE* tricuspid annular plane systolic excursion, *TRPG* tricuspid regurgitation pressure gradient

*P* values refer to the difference between one and any of the remaining three groups

**Fig. 1:**
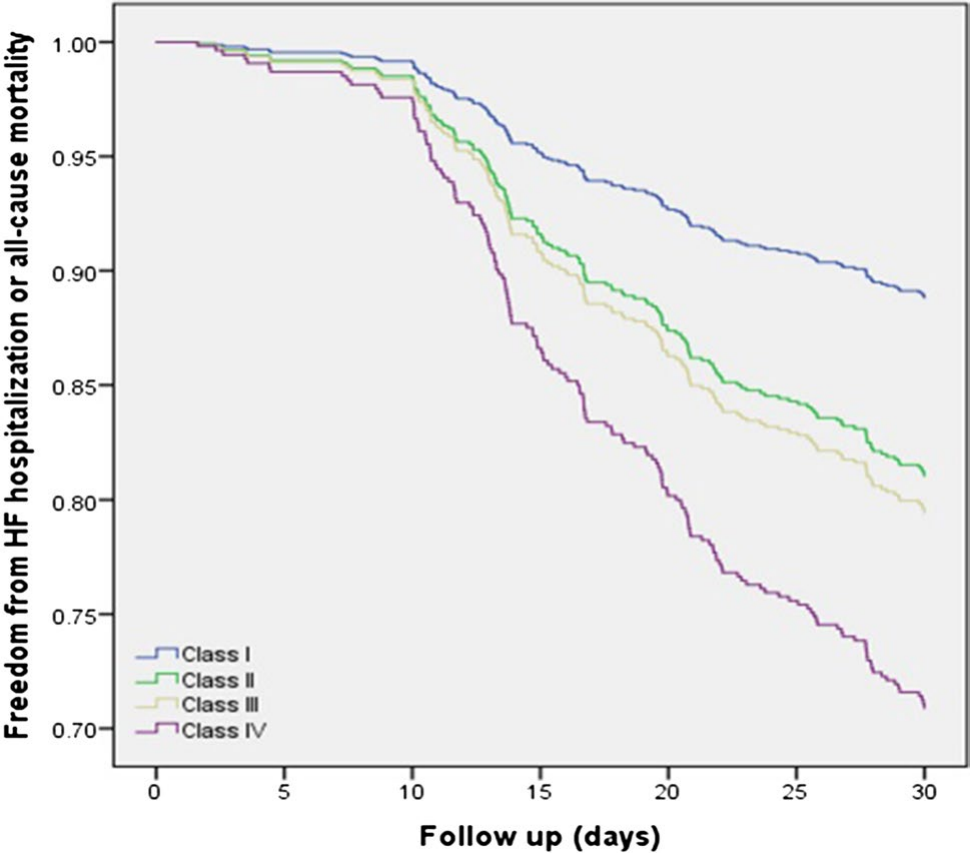
A graded increased risk of 30-day heart failure hospitalization and mortality is shown with the use of the eKillip Class

Compared with eKillip Class I, a graded increase in the combined endpoint of 30-day mortality and rehospitalizations rates was noted (Class II: HR 1.77, CI 0.95–3.33, p = 0.07; Class III: HR 1.94, CI 1.05–3.6, p = 0.034; Class IV: HR 2.9, CI 1.64–5.13, p < 0.001 respectively), which overall persisted after correction for clinical (Class II: HR 1.682, CI 0.9–3.15, p = 0.105; Class III: HR 2.104, CI 1.13–3.9, p = 0.019; Class IV: HR 2.74, CI 1.54–4.85, p = 0.001 respectively) or echocardiographic parameters (Class II: HR 1.92, CI 1.02–3.63, p = 0.045; Class III: HR 1.54, CI 0.81–2.95, p = 0.189; Class IV: HR 2.04, CI 1.1–3.76, p = 0.023 respectively) (Table 3, Fig. 1). Our findings remained statistically significant after excluding patients with severe left sided valvular disease (Supplemental Table 4).

**Table 3:** Univariate and adjusted Cox regression analysis for 30 days of all-cause mortality or HF re-hospitalization according to eKillip class

	Unadjusted			Adjusted for Echo			Adjusted for Clinical		
eKillip class	HR	95% CI	P*	HR	95% CI	P*	HR	95% CI	P*
Class I	Ref			Ref			Ref		
Class II	1.773	0.954–3.296	0.070	1.920	1.015–3.632	0.045	1.682	0.898–3.151	0.105
Class III	1.942	1.05–3.592	0.034	1.542	0.807 – 2.946	0.189	2.104	1.132–3.909	0.019
Class IV	2.903	1.641–5.134	< 0.001	2.037	1.104–3.761	0.023	2.736	1.543–4.851	0.001
Additional analysis:	HR	95% CI	*P*^#^	HR	95% CI	*P*^#^	HR	95% CI	*P*^#^
Class II vs Class IV	1.638	1.097–2.444	0.16	1.144	0.731–1.790	0.557	1.574	1.026–2.413	0.038
Class III vs Class IV	1.494	1.008–2.213	0.045	1.32	0.882–1.975	0.177	1.313	0.875–1.970	0.189

P* values refer to the difference between eKillip Class I and any of the remaining three groups

*P*^#^ values refer to the difference between the 2 groups

Clinical variables: Age, Sex, presence of chronic kidney disease (i.e. glomerular filtration rate < 60ml/min), hypertension, diabetes mellitus, coronary artery disease and AF, hemoglobin levels and guideline-directed HF medication use at admission and at discharge

Echocardiographic variables: LVEF, LV end systolic and end diastolic diameter, E/A ratio, LA size, TRPG, right ventricular size and function

Examining the endpoints separately, compared with eKillip Class I, rates of both 30-day HF hospitalizations (Class II: HR 2.14, CI 1.05–4.37, p = 0.037; Class III: HR 1.99, CI 0.97–4.12, p = 0.062; Class IV: HR 2.05, CI 1.34–5.22, p = 0.005 respectively) and 30-day mortality increased (Class II: HR 1.35, CI 0.41–4.48, p = 0.62; Class III: HR 1.94, CI 0.62–6.11, p = 0.25; Class IV: HR 3.82, CI 1.35–10.8, p = 0.012 respectively) as the eKillip increased (Supplementary Tables 5,6).

## Discussion

HF constitutes a heavy burden to healthcare systems worldwide. Specifically, HF patients suffer high mortality rates and are often readmitted [10]. Consequently, there is an urgent need for developing reliable risk scores which will help with identifying high-risk HF patients, especially those with an acute decompensated HF event. We have demonstrated here that using a simple echocardiographic score, HF patients could be reliably classified into distinct risk groups. The fact that these results were observed within a 30-day period might assist with focusing medical efforts in preventing HF readmissions to high-risk groups of HF patients. Notably, the highest-risk group (eKillip Class IV), which persistently showed statistically significant increased risk for both HF hospitalization and/or mortality, comprised about one-third of the patient population. These important data imply that once appropriately classifying this group as a high-risk one, a focused and dedicated follow-up (and potential interventions) may assist with significantly decreasing overall rehospitalizations rates.

The need for developing clinical, laboratory or imaging tools for identifying high-risk HF patients prone for rehospitalizations and mortality continues to challenge the medical world and different models have been suggested [16–18]. The CHAMPION trial showed that implanting a pressure-monitoring device into one of the pulmonary artery branches provided remote monitoring of pulmonary pressures and induced a significant reduction in rehospitalizations rates [19]. Other devices and models are currently being investigated [20, 21], but all of them require an invasive procedure and dedicated monitoring. Other, simpler methods, such as natriuretic peptides measurement, showed conflicting results as to their ability in predicting post-discharge recurrent hospitalizations and mortality [22–24].

Echocardiography is an accessible, noninvasive, reproducible and reliable tool that is often used to evaluate patients with HF. Studies by the working group on HF of the Italian society of cardiology [8, 9] have shown that different hemodynamic profiles, as assessed by echocardiography, can predict prognosis in HF patient evaluated in the ambulatory setting. Patients with low flow state and elevated filling pressure have the worst outcome.

Consequently, different echocardiographic scores were developed and showed good predictive abilities regarding post-discharge clinical outcomes. Nevertheless, most were either cumbersome or used sophisticated echocardiographic methods [25–27]. For example, Thavendiranathan et al. examined the additive effect of echocardiographic findings to an elaborate risk-prediction tool (the Yale-CORE HF readmission score) and showed that elevated right atrial pressure and left-sided filling pressures added to the predictive ability of the model [27]. In another study, Saito et al. showed that reduced left ventricular global longitudinal strain was associated with worse post-discharge clinical outcomes [26]. Although important, these studies emphasize the need for a straightforward tool which will assist the everyday clinician with identifying HF patients at risk for rehospitalizations and mortality. Our suggested eKillip fits to this description well. The suggested parameters in our model (SVI and E/E') are regularly examined during echocardiography in most facilities. We intentionally chose filling pressure indices which do not require sinus rhythm (i.e. "a wave") and can be applied to the entire HF population including those with AF. Also, the cutoffs which we have used do not significantly differ from the ones used in the routine evaluation of HF [11].

Examining the performance of our model shows that although the categorization of risk did not always reach a statistical significance, it was repeatedly able to categorize the highest-risk group (i.e. eKillip Class IV) appropriately, including following corrections for both clinical and echocardiographic parameters. Notably, this group did not differ from the overall population in other important features such as age, kidney function, the presence of CAD or discharge medication use, emphasizing the added predictive ability of the eKillip.

The pathophysiological basis of our findings emerges from the one which dictated the original Killip score since it captures the fundamental function of the left ventricle. That is, to be able to produce normal perfusion while maintaining normal intracavitary pressures and thereby preventing lung congestion. Numerous trials have demonstrated the importance of SVI and diastolic function on patients' outcomes [28–33]. Furthermore, the predictive ability of both SVI and diastolic function on survival was shown to be superior to LVEF in a recent study conducted in cardiac intensive care patients, emphasizing the importance of perfusion and congestion over systolic function in the acute setting [34].

Our study has a few limitations. First, although large and comprehensive, this is a single-center, retrospective study which did not include the initiation time or the adherence to medical therapy. Second, though echocardiography was done during the index admission, its exact timing might have influenced the results. Third, neither cardiac output (as a surrogate for peripheral perfusion) nor diastolic function (as a surrogate for pulmonary congestion) were fully evaluated. Nevertheless, our aim was to produce a simple tool which will assist with everyday clinical practice and decisions.

In conclusion, we have demonstrated that a simple and reproducible echocardiographic score was able to identify HF patients at risk for 30-day readmissions and mortality. Further studies are needed to test the consistency of our findings in other cohorts. While echocardiographic scores might be found as promising tools for identifying patients at risk, they should not be considered as a substitute for a full echocardiographic assessment.

## Supplementary Information

Below is the link to the electronic supplementary material.

Supplementary file1 (DOCX 40 KB)

## Data Availability

The data underlying this article will be shared on reasonable request to the corresponding author.
