# Supplementary material for: Prediction of recurrent heart failure hospitalizations and mortality using the echocardiographic Killip score
Source: Clin Res Cardiol. 2024 Jun 3;114(5):609–15. doi: 10.1007/s00392-024-02473-8 (PMC12058936; doi:10.1007/s00392-024-02473-8)
Supplement: Supplementary file 1 — Supplementary file1 (DOCX 40 KB) [file 392_2024_2473_MOESM1_ESM.docx]

**Table S4- Univariate Cox regression analysis for 30 days of all-cause mortality or HF re-hospitalization according to eKillip class after exclusion of patients with severe left sided valvular abnormalities**

|  | Unadjusted | | |
| --- | --- | --- | --- |
| eKillip class | HR | 95% CI | P* |
| Class I | Ref. |  |  |
| Class II | 1.704 | 0.909-3.193 | 0.096 |
| Class III | 1.935 | 1.044-3.587 | 0.036 |
| Class IV | 2.593 | 1.454-4.624 | 0.001 |

**Table S5- Univariate and adjusted Cox regression analysis for 30 days of all-cause mortality according to eKillip class**

|  | Unadjusted | | | Adjusted for Echo | | | Adjusted for Clinical | | |
| --- | --- | --- | --- | --- | --- | --- | --- | --- | --- |
| eKillip class | HR | 95% CI | P* | HR | 95% CI | P* | HR | 95% CI | P* |
| Class I | Ref. |  |  | Ref. |  |  | Ref. |  |  |
| Class II | 1.35 | 0.407-4.484 | 0.624 | 1.343 | 0.403-4.47 | 0.631 | 1.119 | 0.333-3.762 | 0.855 |
| Class III | 1.947 | 0.62-6.113 | 0.254 | 1.149 | 0.351-3.761 | 0.819 | 2.068 | 0.654-6.539 | 0.216 |
| Class IV | 3.827 | 1.348-10.864 | 0.012 | 2.312 | 0.782-6.835 | 0.13 | 3.333 | 1.167-9.52 | 0.025 |
| Additional analysis: | HR | 95% CI | P^#^ | HR | 95% CI | P^#^ | HR | 95% CI | P^#^ |
| Class II  vs Class IV | 2.825 | 1.295-6.162 | 0.009 | 2.073 | 0.892-4.816 | 0.09 | 2.966 | 1.321-6.66 | 0.008 |
| Class III  vs Class IV | 1.963 | 0.984-3.918 | 0.056 | 1.989 | 0.96-4.12 | 0.064 | 1.442 | 0.702-2.959 | 0.319 |

P* values refer to the difference between eKillip Class I and any of the remaining three groups.

P^#^ values refer to the difference between the 2 groups.

**Table S6- Univariate and adjusted Cox regression analysis for 30 days of HF hospitalization according to eKillip class**

|  | Unadjusted | | | Adjusted for Echo | | | Adjusted for Clinical | | |
| --- | --- | --- | --- | --- | --- | --- | --- | --- | --- |
| eKillip class | HR | 95% CI | P* | HR | 95% CI | P* | HR | 95% CI | P* |
| Class I | Ref. |  |  | Ref. |  |  | Ref. |  |  |
| Class II | 2.139 | 1.046-4.376 | 0.037 | 2.388 | 1.132-5.034 | 0.022 | 2.311 | 1.121-4.764 | 0.023 |
| Class III | 1.997 | 0.966-4.125 | 0.062 | 1.751 | 0.813-3.774 | 0.153 | 1.982 | 0.955-4.116 | 0.066 |
| Class IV | 2.649 | 1.342-5.229 | 0.005 | 1.977 | 0.944-4.141 | 0.071 | 2.42 | 1.223-4.789 | 0.011 |
| Additional analysis: | HR | 95% CI | P^#^ | HR | 95% CI | P^#^ | HR | 95% CI | P^#^ |
| Class II  vs Class IV | 1.241 | 0.788-1.955 | 0.351 | 0.846 | 0.505-1.419 | 0.527 | 1.068 | 0.652-1.749 | 0.795 |
| Class III  vs Class IV | 1.329 | 0.831-2.127 | 0.235 | 1.127 | 0.7-1.817 | 0.622 | 1.224 | 0.754-1.986 | 0.413 |

P* values refer to the difference between eKillip Class I and any of the remaining three groups.

P^#^ values refer to the difference between the 2 groups.
